# Anterior Obliquity of the Uterus

**Published:** 1845-09

**Authors:** 


*Anterior Obliquity of the Uterus.*—Dr. Pellegrini has published in the Annali Universali di Medicina, the particulars of a case in which labour was complicated by anterior obliquity of the uterus. According to Baudelocque, Velpeau, and others, anterior obliquity of the uterus never presents any obstacle to parturition, and is easily remedied. Baudelocque indeed affirms that the greatest inclination of this kind does not derange the mechanism of parturition, and that he has assisted at many labours which were concluded with facility, where the uterus was inclined so strongly forwards, that the abdomen fell like a sack on the knees. In October, 1840, Dr. Pellegrini was called to a woman who had been in labour for twelve hours—she was ætat. forty; had already borne four children, and for upwards of a year had been subject to gout. The abdomen formed a great sack, which hung down upon the thighs; and, although the woman was in the horizontal position, the fundus of the uterus touched the knees. The woman informed Dr. Pellegrini that her abdomen had begun to fall down in the fourth month, but that it caused her no alarm, for the same thing had occurred in her previous pregnancies without any bad effects. On the present occasion the midwife who had been with her had in vain attempted to replace the uterus: the contractions were strong, and directed from below upward; the membranes had been ruptured several hours. On vaginal examination the head was found presenting at the brim; the uterine orifice was widely dilated, directed to the vertebral column, and the posterior surface of the uterus had become the anterior. It was, of course, impossible for parturition to go on this way, all endeavours to raise the uterus were unsuccessful, and when these attempts were prolonged, the patient was seized with convulsions. Dr. Pellegrini then thought proper to attempt turning: in this he succeeded, but the fœtus was dead, and the mother died four days afterwards of *metro-peritonitis*. Not even when the foetus was extracted could the uterus be replaced, for a large mass of intestines immediately descended on the organ, and kept it still lying on the anterior surface of the thighs.—*Ibid.*

